# Prevalence, Severity, and Treatment Outcomes of Meibomian Gland Dysfunction in Patients With Dry Eye Symptoms at a Tertiary Care Center in South India

**DOI:** 10.7759/cureus.25703

**Published:** 2022-06-07

**Authors:** Jayanti Singh, Yamini Priya, Vivek Bhat

**Affiliations:** 1 Ophthalmology, St. John's Medical College, Bangalore, IND

**Keywords:** hospital-based research, meibomian gland disease, eye irritation, tear film stability, evaporative dry eye, dry eye disease

## Abstract

Background and objective

Dry eye disease (DED) is one of the most common reasons why patients seek eye care. With increasing age, widespread adoption of technology, and environmental changes, its prevalence has been on the rise, and will likely increase further. Meibomian gland dysfunction (MGD) is the most common cause of DED; however, for a variety of reasons, it is currently underrecognized. We aimed to determine the etiology of DED from a sample of patients visiting our center with dry eye symptoms and study the characteristics of those diagnosed with MGD.

Methodology

We conducted this prospective observational study from 2016 to 2018. We included patients with two or more dry eye symptoms and excluded those with systemic conditions or structural issues causing dry eye. Each patient underwent a detailed evaluation of the dry eye, including the Ocular Surface Disease Index (OSDI) questionnaire, Oxford corneal staining, Schirmer test 1, tear film break-up time (TBUT), tear meniscus height, and non-contact meibography. All patients subsequently received appropriate treatment. Patients with MGD were evaluated once again after one month.

Results

We included 250 patients in the study. Their mean (standard deviation) age was 45.3 (16.9) years, and 138 (55%) of them were males. Grittiness and itching were the most common symptoms. MGD was the most common diagnosis, seen in 100 (40%), followed by chronic allergic conjunctivitis. Patients with MGD were more likely to be elderly and had significantly worse DED parameters. Over half of all MGD cases were mild or less severe. With appropriate treatment, all DED metrics improved significantly.

Conclusions

MGD was the most common cause of DED in our sample. Patients with MGD were more likely to be elderly and had more severe DED, consistent with other studies in the literature. With specific treatment of MGD, there was a significant improvement in the patients' condition. DED is a growing ocular health issue that causes great detriment to patients’ quality of life and finances. Our findings support the need for a detailed evaluation and specific treatment of patients presenting with dry eye symptoms.

## Introduction

Dry eye disease (DED) is a growing cause of ocular morbidity and is one of the most common reasons why patients seek eye care. DED broadly refers to a disorder of the ocular surface that results from a compromised tear film. Estimates on its incidence vary, but Indian studies have pegged its prevalence anywhere between 2 and 32% [1,2]. With an aging population, and the increased usage of screens, along with environmental changes, this is only likely to increase [3].

The meibomian glands, which synthesize and secrete crucial lipids that prevent evaporation, are key to this ocular tear film. Meibomian gland dysfunction (MGD) - defined as "a chronic, diffuse abnormality of the meibomian glands, commonly characterized by terminal duct obstruction and/or qualitative/quantitative changes in the glandular secretion" - remains the most common cause of DED worldwide [4]. Despite this, till the last decade, it was commonly overlooked in the ophthalmologic literature [5].

DED, both due to MGD and otherwise, can have a significant impact on patients’ quality of life [6]. In India, however, DED has been understudied. In light of this, we aimed to study the etiologic spectrum of patients with dry eye symptoms due to ocular pathologies, and secondarily, study the prevalence, severity, and treatment outcomes of those with MGD.

## Materials and methods

Study setting and participants

After obtaining approval from the Institutional Ethics Committee (IEC), St. John's Medical College, Bangalore (reference number 314/2016), we conducted this prospective observational study at St. John’s Medical College Hospital, a tertiary care institute in Bangalore, India, from September 2016 to August 2018.

Our inclusion and exclusion criteria are detailed in Table 1. We primarily focused on patients with ocular causes for their DED and excluded those with DED due to systemic causes or structural pathologies.

**Table 1:** Inclusion and exclusion criteria for participants

Inclusion criteria: patients aged ≥18 years with two or more of the following symptoms of dry eye	Exclusion criteria: patients with any one of the following
Foreign body sensation	Contact lens use
Grittiness	History of ocular surgery
Eye irritation	Infectious or acute allergic conjunctivitis
Eye dryness	Stevens-Johnson syndrome
Excessive tearing	Chemical, thermal, or radiation injury
Photophobia	Structural abnormality of one or both eyelids
Eye itching	Alteration of lacrimal drainage system
Eye redness	Acne rosacea

Ophthalmologic evaluation and treatment

After receiving informed written consent, a detailed history was taken and a basic ophthalmologic evaluation including best-corrected visual acuity (BCVA) was done. Following this, each patient underwent a detailed evaluation for dry eye. This included the blink rate, anterior segment examination, slit-lamp examination of the lid margin, the Ocular Surface Disease Index (OSDI), corneal staining, Schirmer 1 test, tear film break-up time (TBUT), tear meniscus height, and non-contact meibography.

We adopted guidelines from the "Tear Film and Ocular Surface Society (TFOS) Dry Eye Workshop (DEWS)" and the "International Workshop on Meibomian Gland Dysfunction, 2010" criteria for meibomian gland expression [4,7]. Based on these, we classified MGD severity as normal, subclinical, minimal, mild, moderate, or severe, and treated it accordingly (Table 2).

**Table 2:** Classification of severity of MGD and treatment for different grades of severity OSDI: Ocular Surface Disease Index; MGD: meibomian gland dysfunction; TBUT: tear film break-up time

	Normal	Subclinical	Minimal	Mild	Moderate	Severe
Symptom frequency and severity	None	Occasional	Sometimes, precipitated by environmental factors	Half of the time, some limitation of activity	Most of the time, frequent limitations of activity	All the time, severe/disabling limitations
OSDI score (0-100)	0	0-12	0-12	13-22	23-32	33-100
MGD grade	Clear	Altered quality only on expression, no gland loss	Minimally altered quality of expressed meibum from scattered glands, minor gland loss	Mildly altered meibum quality, occasional lid margin signs, mild gland loss	Moderately increased viscosity, increased margin vascularity, loss of orifice definition, moderate gland loss	Marked, cicatricial or non-cicatricial margin hyperemia, severe gland loss
Expressed meibum grade (0-24)	0	1-5	6-10	11-15	16-20	21-24
TBUT (seconds)	≥10	7-10	5-7	3-5	1-3	<1
Conjunctival hyperemia	None	None	Minimal	Mild	Moderate	Marked
Oxford corneal staining scale (0-4)	0	0	1	2	3	4
Schirmer 1 score (mm)	≥10	≥10	7-10	5-7	3-5	<3
Treatment	None	Eyelid hygiene, warm compresses	As for subclinical, with artificial tear substitutes, omega-3 fatty acid capsules once a day	As for minimal, with topical azithromycin 1% eye ointment once a day for four weeks	As for mild, with oral tetracycline 250 mg four times a day or oral doxycycline 100 mg twice a day for three weeks	As for moderate, with topical cyclosporine 0.05% eye drops for one month

Statistical analysis

All data were analyzed using SPSS Statistics version 22 (IBM Corp., Armonk, NY). Descriptive statistics are expressed as percentages and means with standard deviation (SD). We used Pearson's chi-squared test to compare the clinical characteristics of patients with MGD and those with DED due to other diagnoses, and paired t-test to compare the dry eye parameters of patients with MGD, before and after treatment. We set the statistical significance level at 0.05 for all analyses.

## Results

Demographic details and ophthalmologic evaluation 

We included 250 patients in this study; 138 (55.2%) of them were males. Ages ranged from 18 to 83 years, with a mean (SD) of 45.3 (16.9) years; 65 (26.0%) were aged between 18-30 years, 51 (20.4%) were aged 60 years or older, while the rest (134, 53.6%) were aged between 31-60 years. 

The most common presenting symptom was grittiness, followed by itching, both reported by over 40% of patients (Table 3).

**Table 3:** Dry eye symptomatology of patients in our sample

Symptom	N (%) (n=250)
Grittiness	110 (44.0%)
Itching	107 (42.8%)
Tearing	91 (36.4%)
Redness	91 (36.4%)
Irritation	74 (29.6%)
Burning	71 (28.4%)
Foreign body sensation	63 (25.2%)
Dryness	34 (13.6%)

Most patients had good BCVA, with 241 (96.4%) patients having BCVA better than 6/18 on the Snellen chart in both eyes. BCVA correlated poorly with dry eye parameters: the correlation coefficient between BCVA and OSDI was 0.394, while that between BCVA and Oxford corneal staining was 0.349.

In our sample, MGD was the most common cause of dry eye symptoms. MGD was seen in 100 (40%) patients, followed by chronic allergic conjunctivitis, which was seen in 75 (30%) patients (Table 4).

**Table 4:** Etiology of DED in our sample MGD: meibomian gland dysfunction; DED: dry eye disease

Cause of dry eye	N (%) (n=250)
MGD	100 (40%)
Chronic allergic conjunctivitis	75 (30%)
Refractive error	37 (14.8%)
Aqueous tear deficiency	24 (9.6%)
Computer vision syndrome	3 (1.2%)
Others	4 (1.6%)

Characteristics of MGD

The characteristics of patients with MGD and those without MGD are summarized in Table 5. Patients with MGD were more likely to be elderly (aged 60 years or more). Further, across all DED parameters, patients with MGD were more likely to have higher abnormal values, compared to those with another diagnosis for DED.

**Table 5:** Characteristics of patients with MGD versus patients with other diagnoses for DED OSDI: Ocular Surface Disease Index; TBUT: tear film break-up time

Characteristic	Value	MGD, n (%) (n=100)	Non-MGD, n (%) (n=150)	P-value
Age (years)	<60	65 (65.0%)	119 (79.3%)	0.01
	≥60	35 (35.0%)	31 (20.7%)	
Sex	Male	58 (58.0%)	80 (53.3%)	0.47
	Female	42 (42.0%)	70 (46.7%)	
OSDI (0-100)	<12	50 (50.0%)	143 (95.3%)	<0.01
	13-32	38 (38.0%)	4 (2.6%)	
	≥33	12 (12.0%)	3 (2.0%)	
TBUT (seconds)	≥10	7 (7.0%)	38 (25.3%)	<0.01
	6-10	64 (64.0%)	111 (74.0%)	
	0-5	29 (29.0%)	1 (0.7%)	
Schirmer 1 test (mm)	≥10	48 (48.0%)	131 (87.3%)	<0.01
	6-10	42 (42.0%)	13 (8.7%)	
	<6	10 (10.0%)	6 (4.0%)	
Meibum score (0-24)	0-10	64 (64.0%)	150 (100%)	<0.01
	11-20	31 (31.0%)	0 (0%)	
	>20	5 (5.0%)	0 (0%)	
Oxford corneal staining (0-4)	0-1	41 (41.0%)	138 (92.0%)	<0.01
	2-3	46 (46.0%)	10 (6.7%)	
	≥4	13 (13.0%)	2 (1.3%)	

Among those with MGD (n=100), 33 (33.0%) patients had subclinical MGD, 24 had minimal MGD (24.0%), 19 (19.0%) had mild, 21 (21.0%) had moderate, and only three (3.0%) had severe MGD.

With appropriate treatment for MGD, we found that all parameters significantly improved, except for Schirmer 1 score (Table 6).

**Table 6:** Results at one-month follow-up after treatment for MGD SD: standard deviation; OSDI: Ocular Surface Disease Index; TBUT: tear film break-up time

Clinical parameter	Pre-treatment mean	Post-treatment mean	Mean (SD) difference	P-value
OSDI (0-100)	6.05	2.52	-3.53 (12.09)	<0.01
Schirmer 1 test (mm)	18.01	18.16	0.15 (7.24)	0.811
TBUT (seconds)	8.49	9.30	0.81 (12.09)	<0.01
Oxford corneal staining (0-4)	0.75	0.39	-0.36 (1.29)	<0.01
Meibum score (0-24)	2.97	1.29	-1.68 (4.92)	<0.01

## Discussion

We found MGD to be the most common diagnosis in our sample of 250 patients with dry eye complaints due to ocular pathologies. Further, we found that patients with MGD were more likely to be elderly, and had more severe DED compared to patients with other diagnoses.

The most common complaint among our patients was itching and grittiness. This slightly differs from prior reports [8,9], but is likely not significant, as symptoms and signs in DED rarely correlate with each other [10]. Further, we did not find any correlation between dry eye severity and the BCVA. Prior reports have indicated that patients with DED have significant functional impairment of the eyes, translating into slower reading and impaired daily functioning [8,11]. In addition, these patients may report blurred vision even if their BCVA is normal per the Snellen chart. Hence, simple visual acuity measurement may not be sufficient in patients with DED, indicating the need for more specific tests in these patients [12].

MGD is the most common cause of DED worldwide. It is reportedly more common in Asian populations, with prevalence rates greater than 60%, compared to around 20% in Caucasian populations [4]. In India, a recent hospital-based study reported a prevalence of 55% [5], and other Indian community-based studies have reported rates of around 30% [13]. A team from Norway recently reported that among their sample of patients with dry eye symptoms, over 90% had MGD. We studied a similar population but found a different result: 40% of our sample had MGD. Larger, community-based studies are needed to arrive at unbiased figures.

Many patients with anatomic features of MGD are asymptomatic, and asymptomatic MGD is reportedly more common than symptomatic MGD [5,14-16]. Those with asymptomatic MGD are at risk of further deterioration to irreversible anatomic changes, resulting in DED. Risk factors include older age, male sex, and certain medications, among others [17]. Consistent with this, we found that patients with MGD were more likely to be older than 60 years, compared to those with another diagnosis. However, we did not find any sex predilection.

We observed that patients with MGD had more severe DED, compared to other DED patients: 50% had an OSDI score of 13 or more, compared to only about 5% of those with other diagnoses. This is consistent with our clinical experience. Further, it is consistent with findings from Norway and China, where researchers reported a significant association between symptom burden (OSDI score of 13 or more) and the presence of MGD [14,18].

In our sample, while still significantly worse than patients without MGD, almost half of those with MGD had a Schirmer 1 test result of 10 mm or more, i.e., a normal result. This is consistent with prior reports [14], indicating that tests for aqueous deficiency may not be an objective metric in MGD. This is expected, given that evaporation of the tear film is the cause of DED in MGD. On appropriate treatment, all patients showed remarkable improvement. Only the Schirmer 1 score did not show a significant difference, further supporting our earlier conclusions.

Our study has a few limitations. Our sample consisted of patients visiting a single hospital with dry eye symptoms. Many patients had visited other centers before finally coming to ours due to persistent complaints. In addition, we excluded patients with systemic diseases that can cause dry eye symptoms. Thus, our sample was probably skewed towards greater severity and likely resulted in an overestimation of the prevalence of MGD. Further, we used metrics such as the OSDI to quantify the severity of DED. These metrics have been validated in Western populations, but may not be as accurate in Indian patients [5]. Finally, we did not assess the volume or quantity of secreted lipids in the patients' tear films, which may have provided us with more information [5]. However, we used a relatively large sample, which was balanced in terms of age and sex. Further, we ensured a comprehensive evaluation of each patient, which, we hope, made up for some of the limitations in individual metrics.

DED is rapidly on the rise worldwide and can result in significant economic burdens and impaired quality of life for patients [19]. Yet, in many cases, patients, unfortunately, do not receive a detailed evaluation and are given symptomatic treatment with lubricating eye drops alone. While MGD and DED overlap, they are unique pathologies with different risk factors, etiology, and pathophysiology [20]. This justifies the need for an in-depth evaluation of DED, to ensure adequate treatment.

## Conclusions

In our sample of patients with ocular causes for dry eye complaints, MGD was the most common diagnosis. Patients with MGD were more likely to be older and have more severe DED. With specific treatment, most of these patients showed significant improvement, emphasizing the need for a detailed evaluation and tailored management of patients with symptoms of DED.
